# The effect of etidronate on choroidal neovascular activity in patients with pseudoxanthoma elasticum

**DOI:** 10.1371/journal.pone.0240970

**Published:** 2020-10-20

**Authors:** Sara Risseeuw, Redmer van Leeuwen, Saskia M. Imhof, Pim A. de Jong, Willem P. Th. M. Mali, Wilko Spiering, Jeannette Ossewaarde–van Norel

**Affiliations:** 1 Department of Ophthalmology, University Medical Center Utrecht, Utrecht University, Utrecht, The Netherlands; 2 Department of Radiology and Nuclear Medicine, University Medical Center Utrecht, Utrecht University, Utrecht, The Netherlands; 3 Department of Vascular Medicine, University Medical Center Utrecht, Utrecht University, Utrecht, The Netherlands; PLOS ONE, UNITED KINGDOM

## Abstract

**Aim:**

To assess the effect of the bisphosphonate etidronate on choroidal neovascular (CNV) activity in patients with pseudoxanthoma elasticum (PXE).

**Methods:**

This is an ancillary study in a single center, randomized, double-blind placebo-controlled trial (RCT) in which 74 patients with PXE were assigned to either one-year etidronate or placebo treatment. Spectral domain optical coherence tomography (SD-OCT) imaging and color fundus photography were performed every three months for one year and were systematically assessed on signs of CNV activity.

**Results:**

In the etidronate group, 11 (30%) of the patients had CNV activity at baseline, compared to 25 (67%) of the patients in the placebo group (*P* = 0.005). The proportion of eyes with CNV activity during the study ranged from 18–33% in the etidronate group and 42–56% in the placebo group and no significant difference in improvement or worsening of CNV activity was found (*P* = 0.168). Using a generalized mixed model for repeated measures, there was a protective effect of etidronate in crude analysis (RR 0.86, 95% CI 0.75–0.98) that disappeared when adjusting for baseline CNV activity (RR 0.97, 95% CI 0.84–1.13).

**Conclusion:**

In this post-hoc RCT analysis we did not observe a protecting or deteriorating effect of etidronate on CNV activity in patients with PXE after adjustment for baseline CNV.

## Background

Choroidal neovascularization (CNV) is a common sight-threatening complication in chorioretinal diseases such as age-related macular degeneration (AMD), pathologic myopia and pseudoxanthoma elasticum (PXE) [1, 2]. Despite prompt treatment with anti-vascular endothelial growth factors (anti-VEGF), irreversible retinal damage and scarring often occurs [3].

In PXE, bi-allelic mutations in the *ABCC6* gene cause ectopic mineralization in the skin, vasculature and in Bruch’s membrane (BM) of the eye [4]. The latter results in BM breaks, so-called angioid streaks, which allow CNV to arise [2]. These cause visual impairment at a relatively young age [5]_._ Up to now, no causal treatment for PXE exists. Ectopic mineralization in PXE is likely caused by low levels of inorganic pyrophosphate (PPi), an endogenic ectopic mineralization inhibitor [6]_._ Bisphosphonates, which are synthetic analogs of PPi, might therefore have a beneficial effect on ectopic mineralization. To further study the effect of bisphoshonates on vascular calcification in patients with PXE, a randomized placebo-controlled double-blinded trial was conducted at the University Medical Center Utrecht (‘Treatment of Ectopic Calcification in PXE’ (TEMP) trial) [7].

At the start of the trial data on possible effects of bisphosphonates on CNV activity were conflicting [8–11]. Treatment of CNV due to AMD or myopia with alendronate was associated with better visual outcome in some studies [8, 9]_._ However, a higher risk of CNV in AMD was found in users of oral bisphosphonates in a case-control study [11]_._ This might be explained by the pro-inflammatory properties of bisphosphonates [12]_._ These inconclusive findings gave reason to closely monitor the eye disease and CNV activity in participants of the TEMP trial. Earlier, we reported that users of etidronate had fewer subretinal neovascularization events. These were defined as indications to start or intensify anti-VEGF injections and considered to be serious adverse events (SAE). However, we did not directly and systematically measure the SAE on imaging [7].

In this post-hoc analysis of the TEMP trial, we aim to assess the effect of the bisphosphonate etidronate on CNV activity as measured by optical coherence tomography (OCT) imaging in patients with PXE.

## Methods

### Study design and population

For this study, data was used from a single center, randomized, double-blind placebo-controlled trial to study the effect of etidronate on ectopic calcification in patients with PXE (Dutch Trial Register, number NTR5180) [7]. This study was performed at the University Medical Center in Utrecht and the medical ethics committee approved the protocol (METC number 15/522). All participants provided written informed consent. In total, 74 patients were included who met the clinical criteria for PXE as proposed by Plomp et al. [13] (patients should have at least two of the following features: characteristic skin lesions, eye involvement and/or genetic confirmation of PXE). An inclusion criterium was the presence of calcification in the femoral arteries, as this was the main outcome parameter of the trial. Retinal abnormalities were not taken into account as in- or exclusion criterion. Patients were randomized to either a daily dose of 20 mg/kg of etidronate during 2 weeks, followed by a 10-week period without treatment, or an identical regimen with placebo during 12 months. Further details on selection, randomization and treatment of patients can be found in a previous report on this trial [7].

### Assessment of outcome

All participants were seen at baseline and after 3, 6, 9 and 12 months follow-up. At each visit, imaging included color fundus photography (FF450 plus, Carl Zeiss Meditec AG, Jena, Germany) and spectral domain OCT imaging (SD-OCT) (Spectralis HRA-OCT, Heidelberg Engineering, Heidelberg, Germany). SD-OCT imaging consisted of 25 horizontal B-scans of 6 mm length and interscan distance of 250 μm, so that the major part of the macular area was included. Eye-tracking technology allowed the B-scans to be positioned at the exact same anatomical location at each follow-up visit. At baseline and 12 months follow-up, best-corrected visual acuity (BCVA) was measured using Early Treatment of Diabetic Retinopathy Study (ETDRS) letter charts [14]. Clinical monitoring of CNV and anti-VEGF treatment was performed by the referring ophthalmologist according to current guidelines, independent from study visits. In case of concerns regarding CNV activity at study visits, the ophthalmologists involved in the trial consulted the referring ophthalmologists promptly.

Information on anti-VEGF treatment in the 12 months before and during the trial was systematically collected. This information consisted of the date and type of injected drug per eye (bevacizumab, ranibizumab or aflibercept), in case the patient was treated.

**Fig 1:**
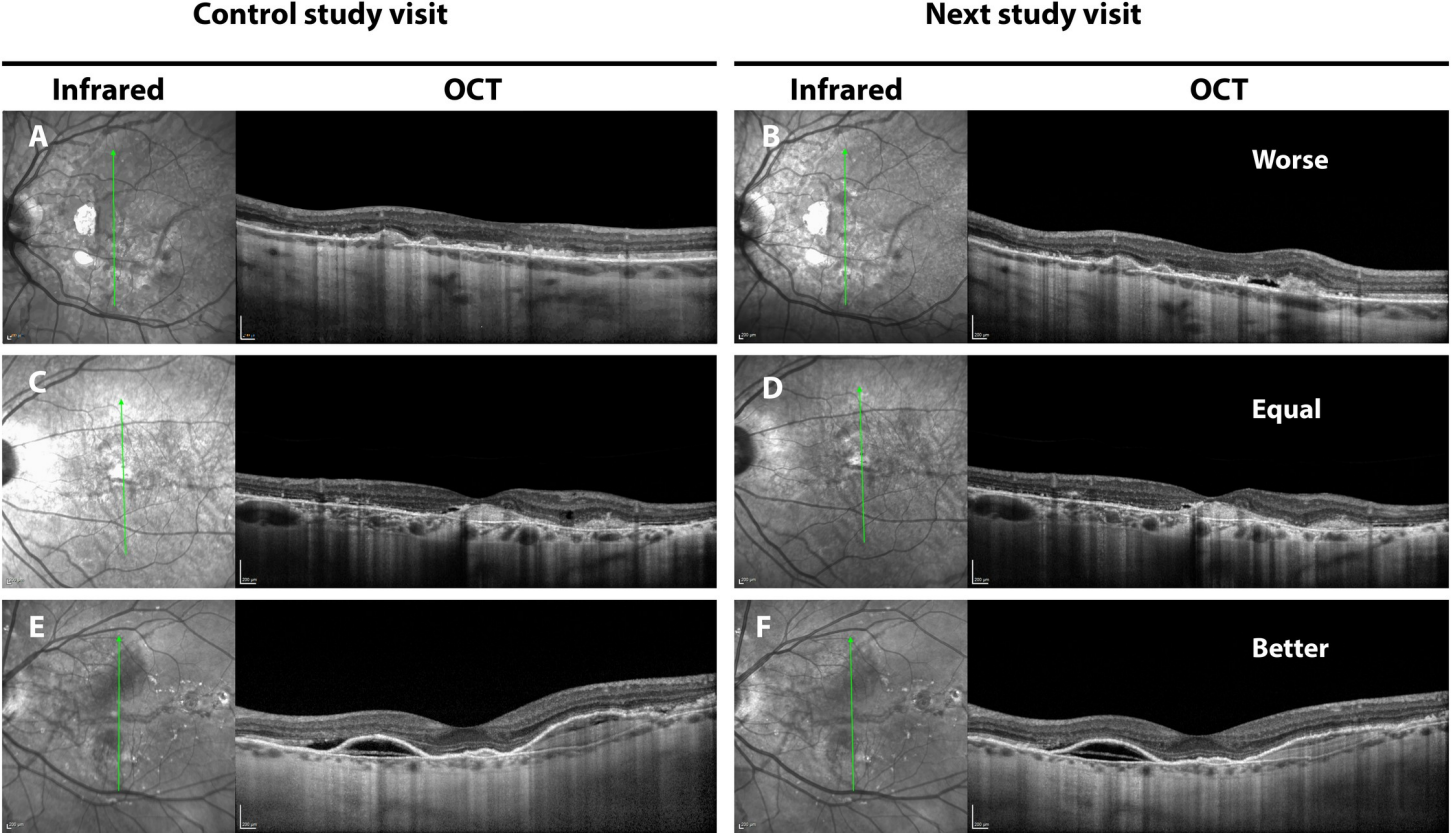
Changes in CNV activity. Optical coherence tomography (OCT) imaging of changes in CNV activity in patients with pseudoxanthoma elasticum with corresponding infrared image. The time interval between the study visit and the next visit is three months. The upper images (**A** and **B**) show an increase of subretinal fluid and outgrowth of the CNV complex and illustrates a worsening of CNV activity. In the middle (**C** and **D**), the CNV is inactive and stable. Below (**E** and **F**) a decrease of subretinal fluid is seen, illustrating an improvement of CNV activity.

For the purpose of this post-hoc study, assessment of imaging was performed blinded to study arm by three individual graders (SR, JOvN, RvL), by systematically grading all SD-OCT imaging and fundus color photographs of both eyes at each visit. Both the presence and a change in amount of intra- or subretinal fluid (compared to the previous investigation) were scored. Unfortunately, fluorescence angiography was not available to detect leakage of CNV. We believe that this should not be a problem, because angiography does not add information regarding CNV activity in PXE patients in most cases [15]. CNV activity was diagnosed if at least one of the following criteria was present: 1) hemorrhage in posterior pole on color fundus photography and/or 2) one or more intraretinal cysts of >50 μm diameter and/or subretinal fluid of >200 μm width, with signs of a CNV nearby, and/or 3) obvious growth of a subretinal neovascular complex. In this paper, for simplicity, this suspicion of neovascular activity is called ‘neovascular activity (CNV activity)’. Change in the intra- or subretinal fluid, which we defined to be signs of CNV activity, was graded as ‘better’, ‘equal’ or ‘worse’, based on an overall conclusion of all 25 B-scans as compared with the previous OCT-scan (as illustrated in Fig 1) In case of discrepancy, the major vote of the three graders was taken for both CNV activity and the change in CNV activity. Furthermore, the macular phenotype was assessed according to previously reported criteria and the eyes were categorized in four groups: atrophic, CNV lesions, mixed or no CNV or atrophy [5]_._

### Statistical analysis

BCVA was converted to the logarithm of the minimum angle of resolution (logMAR) for statistical purposed. Descriptive data are presented as numbers with percentage (%), mean with standard deviation or median with interquartile range, depending on the distribution of the values. At baseline, data are presented at patient level, to illustrate the differences between the etidronate group and the placebo group. For descriptive data regarding CNV activity, data are presented per eye. Differences between the groups were tested with the chi-square test for categorical variables, students’ t-test for normal distributions or Mann-Whitney U test for non-parametric distributions. We considered a *P* value lower than 0.05 statistically significant.

Regression analysis was used to investigate associations between treatment and outcome. A mixed effects Poisson regression model for repeated measures was used to analyze the association between etidronate and the presence of CNV activity per eye [16]_._ In the crude analysis, treatment status and time of visit were included as fixed effects. The individual patient and the left or right eye (nested within the patient) were considered random effects. In the adjusted analysis, baseline CNV activity of the concerning eye was included as a fixed effect. For the effect of etidronate on change in BCVA, we used the difference in ETDRS letters between baseline and 12 months follow-up in a linear regression model.

Interobserver consistency was measured using the percentage of agreement for all three graders on a random subset of 74 eyes [17].

Statistical analysis was performed with R version 3.4.1 (www.R-project.org). The packages ‘irr’ and ‘lme4’ (version 1.1–15) were used for agreement and mixed effects analysis, respectively.

## Results

### Baseline data

In total, 74 patients were included. One patient in the etidronate group discontinued treatment after six months due to hypersensitivity complaints but remained in the study. One patient in the placebo group discontinued participation in the trial after three months due to uveitis following an anti-VEGF injection [7]_._ In four eyes (all without SAE), comparison of OCT scans was impaired by low quality and the data from these scans were excluded from the presented results.

The mean age was 57 years and 51% was male. The macular phenotype and the BCVA were similar in the placebo and intervention group. However, there was a baseline imbalance regarding CNV activity (Table 1). In the placebo group, 25 patients (67.6%) had CNV activity in one or both eyes, compared to 11 (29.7%) in the etidronate group (*P =* 0.005). This baseline imbalance was not seen in the median number of anti-VEGF injections 3 months before the start of the trial (2.0 in the etidronate group vs 2.5 in the placebo, *P* = 0.848). Comparison of the median number of anti-VEGF injection 6 and 12 months before the trial yielded similar results.

**Table 1:** Baseline ocular characteristics of participants.

	Etidronate	Placebo	*P*value
	n = 37	n = 37	
Age (mean ± SD)	56.9 ± 8.6	57.0 ± 8.0	0.956*
Male (%)	19 (51)	19 (51)	0.999
**Ocular phenotype**			
Macular phenotype: right eye (%)			0.156**
*Atrophy*	1 (3)	7 (19)	
*CNV*	11 (30)	10 (27)	
*Mixed*	15 (41)	13 (35)	
*No CNV or atrophy*	10 (27)	7 (19)	
Macular phenotype: left eye (%)			0.220**
*Atrophy*	0	2 (5)	
*CNV*	10 (27)	15 (41)	
*Mixed*	18 (49)	15 (41)	
*No CNV or atrophy*	9 (24)	5 (14)	
BCVA (in logMAR) of right eye (median [IQR])	0.32 [0.00–1.36]	0.34 [0.10–1.18]	0.935***
BCVA (in logMAR) left eye (median [IQR])	0.56 [0.04–1.52]	0.70 [0.07–1.32]	0.816***
Best BCVA (in logMAR)	0.14 [0.00–0.88]	0.26 [0.06–0.96]	0.681***
Worst BCVA (in logMAR)	1.18 [0.14–1.60]	1.18 [0.18–1.44]	0.762***
**Neovascular activity at baseline**			
Activity per patient (%)			0.005***
*No CNV activity*	26 (70)	12 (32)	
*One eye*	9 (24)	19 (51)	
*Both eyes*	2 (5)	6 (16)	
CNV activity in right eye (%)	7 (19)	14 (38)	0.122***
CNV activity in left eye (%)	6 (16)	17 (46)	0.012***
**Patient treated with anti-VEGF before the start of the trial?**			
Three months before start of trial	14 (38)	14 (38)	0.999***
*Number of injections (median [IQR])*^*a*^	2.0 [2.0–3.0]	2.5 [2.0–3.0]	0.848**
Six months before start of trial	16 (43)	15 (41)	0.999**
*Number of injections (median [IQR]*^*a*^^*a*^	4.0 [2.8–6.0]	4.0 [3.0–5.5]	0.826***
12 months before trial	17 (46)	17 (47)	0.999**
*Number of injections (median [IQR])*^*a*^	8.0 [5.0–11.0]	9.0 [5.0–12.0]	0.809***

Abbreviations: SD; standard deviation, BCVA; Best corrected visual acuity, logMAR; logarithm of the minimum angle of resolution, CNV; choroidal neovascularization, ETDRS; Early treatment of Diabetic Retinopathy Treatment Study group, IQR; interquartile range, VEGF; vascular endothelial growth factor.

a. The number of injections is presented only for the patients that received anti-VEGF treatment prior to the trial.

* Differences tested with students t-test.

** Differences tested with chi-square test.

*** Differences tested with Mann-Whitney U test.

### Proportion of and change in CNV activity

**Fig 2:**
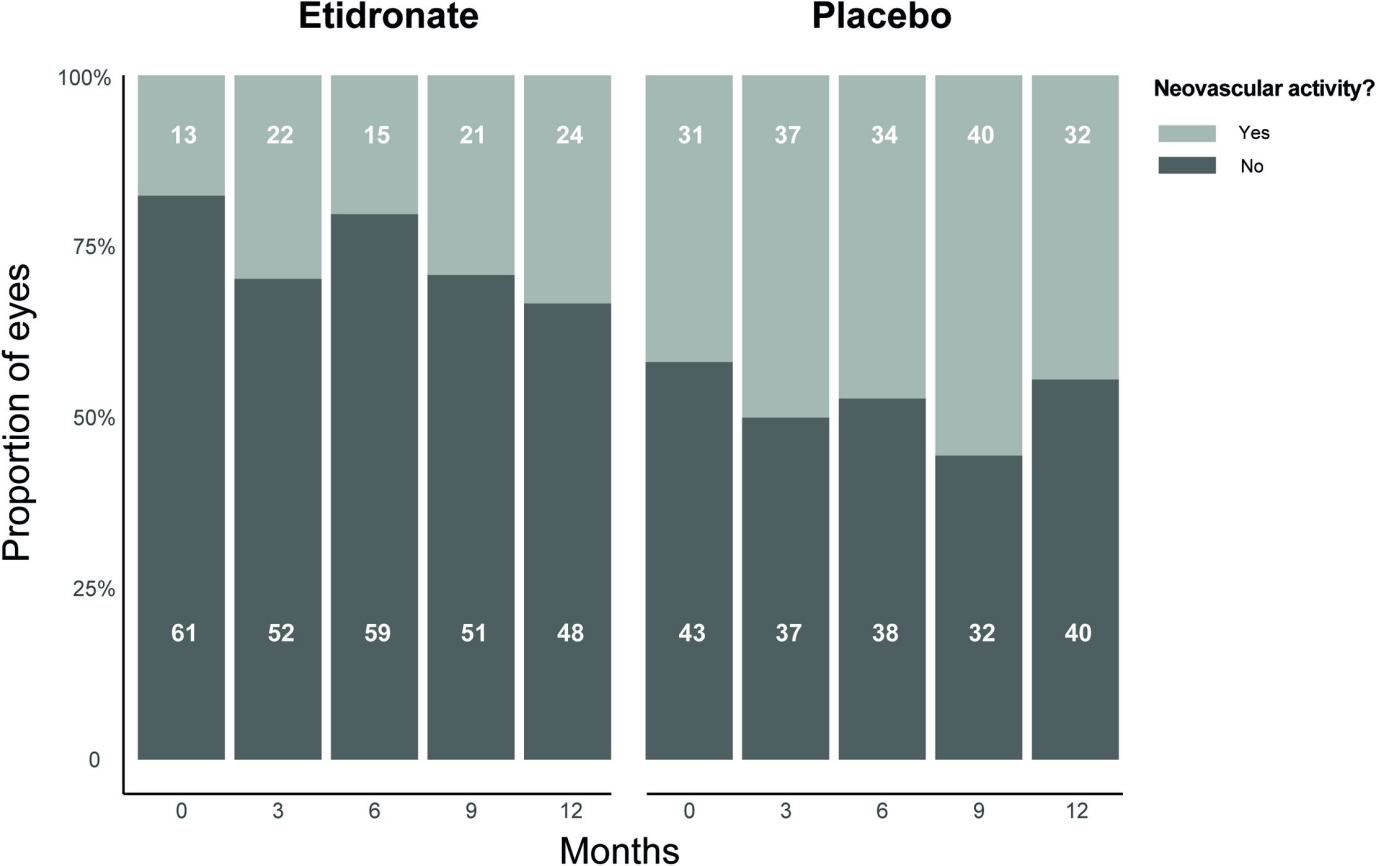
Proportion of eyes with CNV activity^a^. CNV; choroidal neovascularization. The white numbers represent the absolute number of eyes per group. *a*. CNV activity was diagnosed if at least one of the following criteria was present: hemorrhage in the posterior pole on color fundus photography, and/or intra- or subretinal fluid with signs of CNV nearby, and/or growth of a CNV complex.

**Fig 3:**
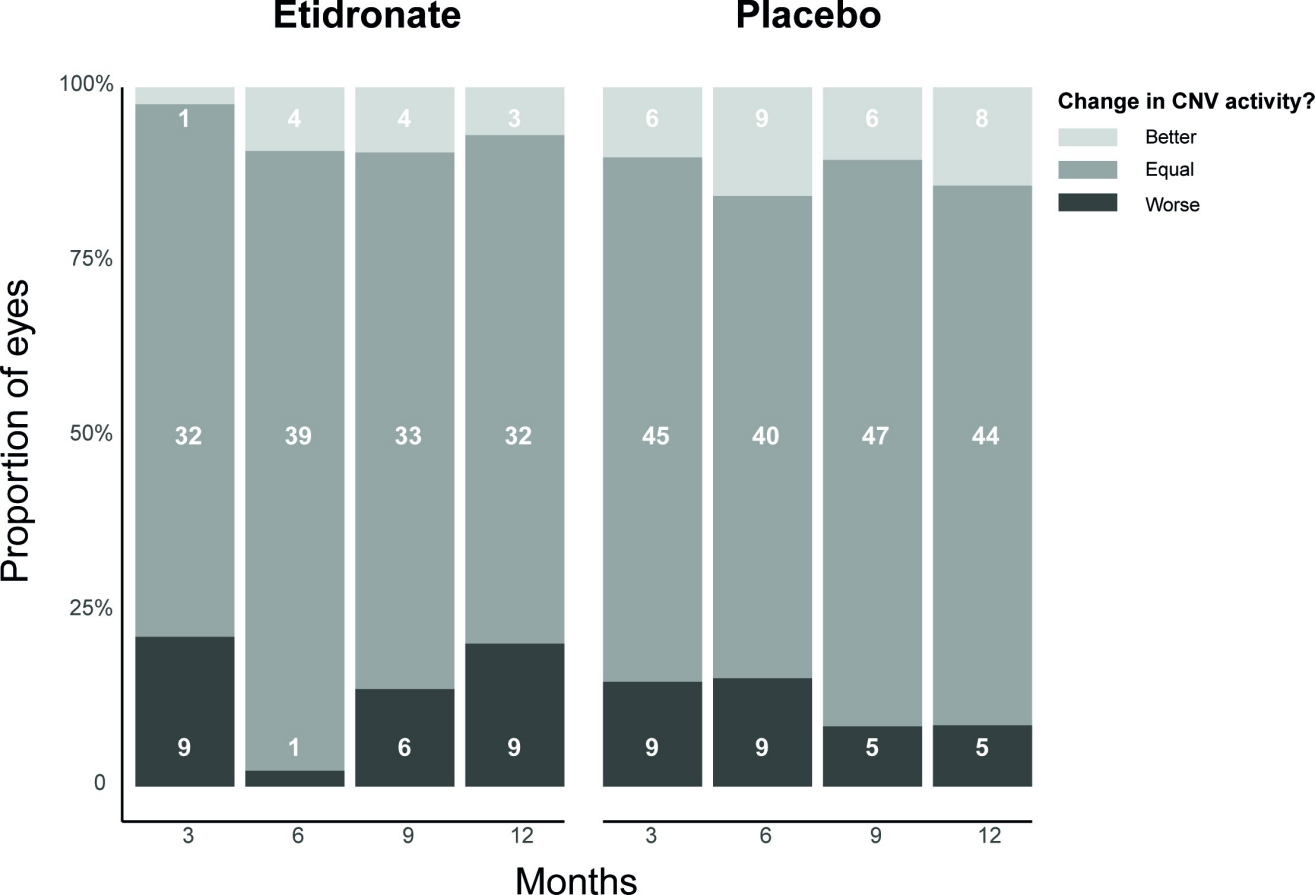
Change in CNV activity in patients (n = 52) with at least one neovascular active period in one or both eyes during the trial. ^**a**^ CNV; choroidal neovascularization. The white numbers represent the absolute number of eyes per group. *a*. Change in CNV activity was based on a subjective assessment of all B-scans compared to the previous examination.

The proportion of eyes with CNV activity in both the etidronate and the placebo group during the 12 months of the trial is presented in Fig 2. Of the two patients that discontinued treatment, only the available imaging during the treatment is presented. The proportion of eyes with CNV activity ranged from 13–24 (18–33%) in the etidronate group and 31–40 (42–56%) in the placebo group. Out of all these patients having CNV activity, only ten patients had clinically relevant CNV activity in the TEMP trial, requiring immediate start or intensifying of anti-VEGF treatment. Of all patients, 52 (69%) had at least one neovascular active episode during the trial (22 patients in the etidronate and 30 patients in the placebo group). Of those patients, the change in CNV activity per eye is presented in Fig 3. CNV activity worsened in 1–9 (2–21%) of the eyes in the etidronate group and 5–9 (8–16%) in the placebo group, when compared to the previous study visit. CNV activity improved in 1–4 (2–9%) of the eyes in the etidronate group and 6–9 (10–16%) in the placebo group, when compared to the previous study visit. Overall, the change in CNV activity was similar between the etidronate group and the placebo group (*P* = 0.168 (chi-square test)). The percentage of agreement was 73% for presence of CNV activity and 69% for change in CNV activity.

### Effect of treatment

The median number of anti-VEGF injections administered during the TEMP trial to patients having at least one episode with CNV activity (n = 52), was similar between the etidronate and placebo group (placebo group (n = 30) 3.5 (IQR 0–8) versus etidronate group (n = 22) 0 (IQR 0–6), *P* = 0.223, Mann-Whitney U test). Confining to patients who had at least one injection (n = 28), there was also no difference: median of 8 (IQR 6–9) injections in the placebo group (n = 18) compared to 6 (IQR 4–9) in the etidronate group (n = 10) (*P* = 0.485, Mann-Whitney U test).

Nearly all events occurred in the second half year of the trial, thereby possibly increasing the number of injections. Considering the number of injections in the first 6 months of the TEMP trial, no significant difference was found: the placebo group (n = 13) received a median of 4 injections (IQR 3–5) compared to 3 (IQR 2–4) in the etidronate group (n = 10) (*P* = 0.271, Mann-Whitney U test).

The effect of etidronate on presence of CNV activity was analyzed using repeated measurements in a mixed effects Poisson regression model. Crude analysis showed a protective effect of etidronate (relative risk (RR) 0.86, 95%CI 0.75–0.98) on CNV activity. When correcting for baseline activity in multivariable analysis, this effect disappeared and was no longer statistically significant (RR 0.97, 95%CI 0.84–1.13). Baseline CNV activity was an indicator of CNV activity during follow-up (RR 1.60, 95%CI 1.38–1.85).

The median BCVA in logMAR at twelve months in the etidronate group was 0.36 (IQR 0.03–0.1.38) in the right eye and 0.67 (0.10–1.47) in the left eye. In both eyes, the BCVA was not significantly different from the placebo group (0.63 (IQR 0.11–1.20) in the right eye and 0.74 (IQR 0.13–1.28) in the left eye (*P* = 0.906 and *P* = 0.977, respectively (Mann-Whitney U test). In unadjusted linear regression analysis, etidronate was not associated with a change in BCVA (β -0.03 (95%CI -0.09–0.03) in the right eye, β -0.01 (95%CI -0.04–0.05) in the left eye).

## Discussion

We found no protecting or deteriorating effect of the bisphosphonate etidronate on the activity of CNV in patients with PXE after adjustment for baseline CNV activity in this randomized, placebo-controlled, double-blind trial.

This post-hoc study provides additional evidence regarding the effect of bisphosphonates on CNV in a monogenetic disorder. So far, study results regarding the effect of bisphosphonates on CNV were conflicting. In a large observational study, the use of the bisphosphonates alendronate, ibandronate and risedronate was associated with an increased risk of neovascular AMD [11]. However, due to the non-randomized design and the disproportionality analysis this study was not able to correct for confounding due to age-related comorbidities. Also, change in CNV activity was not assessed in relation to the registered drug use. Another prospective non-randomized trial in 40 eyes with CNV due to AMD or myopia found a beneficial effect of alendronate on the lesion size and visual acuity after six months [8]. However, this study was uncontrolled and prone to selection bias, since patients were recruited who declined anti-VEGF treatment and may have had less severe CNV. Another study retrospectively observed eyes with myopic CNV, among which 15 eyes included in the previous study [8, 9]. Patients treated with alendronate maintained vision after two years in contrary to the untreated group, and this effect was comparable to the effect of photodynamic monotherapy. The small sample size and risk of selection bias made it difficult to draw a reliable conclusion. The authors suggested that bisphosphonates may have anti-angiogenic properties, next to pro-inflammatory properties. CNV activity responds to intravitreal anti-VEGF therapy, not only in pathological myopia and AMD, but also in PXE [18]. Therefore, if the bisphosphonate etidronate would have anti-angiogenic properties, this might also be expected to observe in PXE patients. In an *in vitro* study in mice with CNV, alendronate inhibited VEGF gene expression [10]_._ In contrast, clodronate showed no difference in gene expression *in vitro* [10]. Another study in human RPE cells found that both etidronate and alendronate reduce expression of angiogenic factors, among which basic fibroblast growth factor [12]. In both studies, the observed effect was dose-dependent. Considering the limited evidence regarding anti-angiogenic factors, the potential opposing effect of pro-inflammatory properties, and the null effect in our post-hoc analysis in a randomized double-blind intervention trial, the role of bisphosphonates in retinal angiogenesis seems to be negligible.

In this cohort of PXE patients, the proportion of eyes with CNV activity during a one-year observation period ranged from 18 to 56%. Since this is the first study to report on the occurrence of CNV activity in PXE, it is difficult to compare these percentages to the natural course of the disease. However, we think that actual CNV activity is overestimated in this study. Firstly, participants of this trial were selected based on vascular calcification. Since PXE is a progressive disease, the study cohort represents older PXE patients who suffer more often from CNV than younger patients [19]. Secondly, according to the aforementioned criteria, all intraretinal cysts with signs of a nearby CNV were graded as CNV activity. Especially in end stage disease, intraretinal cysts may represent atrophy rather than active CNV. Since fluorescein angiography was not available at all examinations, this imaging tool was not included in the CNV criteria. We considered our clinical approach to be as objective as possible, since the macular pathology in PXE influenced imaging quality in such a way that volumetric measurements regarding fluid or CNV complex were not possible in a major part of the OCT-scans.

In the main paper of the TEMP trial, we reported a statistically significant lower incidence of subretinal neovascularisation events during the study in the etidronate group compared to the placebo group (1 event versus 9 events, respectively) [7]. The difference with the results reported in the present study can be explained by a different outcome definition and method of analysis. In the main paper, the definition of a subretinal neovascular event was based on clinical relevance, necessitating immediate therapeutic intervention with anti-VEGF and all events were reported as SAE’s to the Medical Ethics Committee and the Data Safety and Monitoring Board. To assess the subretinal neovascularization events, we used all available information from medical records regarding CNV activity, with the aim to establish safety since we did not know the possible effects of etidronate on CNV activity. Also, the results presented in the paper of the TEMP trial were based on differences between the etidronate group and the placebo group, unadjusted for baseline CNV activity. In contrast, in the present study, our aim was to investigate all changes in CNV activity blinded for treatment status, based on the SD-OCT imaging and fundus photography throughout the study, and also including subtler changes. We used a mixed model approach to incorporate the multiple study visits and to control for baseline CNV activity, instead of only testing differences between treatment groups. Univariable analysis showed that the use of etidronate was associated with a lower incidence of CNV activity. When correcting for baseline CNV activity in a mixed effects model for repeated measures, this association diminished and was no longer statistically significant. Therefore, we conclude that baseline CNV activity is an important predictor of both change in CNV activity and of subretinal neovascular events, as defined in the previous report.

The dosage of etidronate used in this trial is different from standard care. Patients received a dose of etidronate which is at least four times higher than dosing for regular osteoporosis treatment. However, since the effect of etidronate appears to be dose-dependent [12, 20], we hypothesize that this high dose would only increase the effect size, if there was any. Thus, given that no effect was found on either worsening or improvement of CNV activity, we assume that a common dosage of bisphosphonates would not change these findings. We do not know whether the type of bisphosphonates is relevant regarding anti-angiogenic effects. Etidronate belongs to the group of non-aminobisphosphonates, which is less potent in the inhibition of bone resorption and prevention of fractures than the newer aminobisphosphonates, such as alendronate. Etidronate acts predominantly on the mineralization process, while aminobisphosphonates inhibit activity of osteoclasts [21, 22]. Another difference between these two groups is that aminobisphosphonates produce more pro-inflammatory cytokines [23]. Both types of bisphosphonates are associated with reduced angiogenesis, but the clinical relevance in ophthalmology is unclear [9, 24, 25]. Also, it is unclear whether the effect of varying cytokine levels, as influenced by different bisphosphonates, on the activity of CNV is clinically relevant. Therefore, generalization of these findings to aminobisphosphonates is difficult. However, it is important to realize there is substantial evidence that etidronate can halt systemic calcification [7, 26, 27]. The inhibition of calcification might be another mechanism, independent of angiogenesis and inflammation, by which etidronate may be beneficial for ophthalmological disease in PXE. To determine the effect of etidronate on BM calcification, further investigation with methods that visualize the calcification process at Bruch’s membrane are warranted and require a longer follow-up to show an effect on visual acuity, the area of retinal atrophy and number of anti-VEGF injections.

There are some limitations to be addressed. This study was a post-hoc analysis of a trial that was not powered on CNV activity as an outcome measure. Therefore, the sample size is relatively small and the imaging was not optimized to quantify CNV activity, which likely reduced statistical power. The criteria to grade CNV activity and change in CNV activity in PXE based on OCT have been developed by the authors, since the macular pathology made it too difficult to apply quantitative measures on imaging. The interobserver consistency of grading the CNV activity and change in activity was not high, which stresses the difficulty of assessing CNV activity in PXE patients. Subtle CNV activity is sometimes difficult to distinguish from natural variation in the amount or size of residual intraretinal cysts or subretinal fluid. We anticipated that the agreement would not be perfect due to complicated assessment of the extensive pathology, we accounted for this by using the major vote. By using the major vote instead of one grader, we have decreased the risk of misclassification bias, which could reduce the power of the associations. Even though the CNV assessment has not been validated yet, the randomized, placebo-controlled design and blinded grading make these assessments comparable between both groups. Furthermore, the schedule of the anti-VEGF injection treatment was determined by the referring ophthalmologist and was independent of the study visits, thus the time interval between study visits and injections was not standardized. However, the referring ophthalmologists were blinded for treatment status, therefore the time interval from anti-VEGF injection to study visit varies at random. Also, the analysis of the number of anti-VEGF injections during the TEMP-trial showed that the possibility of longer intervals since the visit with their referring ophthalmologist in the placebo group cannot explain the higher number of neovascular events in the placebo group in the TEMP trial. A limitation of our analysis using repeated measures is that we did not use robust errors variance in our model, which can be considered as a modification of a Poisson regression for binomial data [28]. Therefore, we performed a sensitivity analysis using logistic regression. This showed similar crude and adjusted effects. However, this analysis yielded high odds ratios and was therefore difficult to interpret. Lastly, the generalizability of these findings to patients with CNV secondary to AMD or pathologic myopia is unknown. Perhaps bisphosphonates act through PXE specific pathologic processes, i.e. calcification of Bruch’s membrane. This hypothesis has still to be proven. If bisphosphonates have a direct anti-angiogenic effect, it seems plausible that a similar association would be observed in the activity of CNV due to AMD and myopia.

In conclusion, in this randomized, placebo-controlled, double-blind trial, no protecting or deteriorating effect of the bisphosphonate etidronate on CNV activity in patients with PXE was found after adjustment for baseline CNV activity. Based on this high-dosing trial, it seems safe to prescribe bisphosphonates to PXE patients and further research on long-term effects and on Bruch’s membrane calcification is warranted.
